# Eighteen Years of Medical Oncology in Morocco: A Bibliometric Evaluation

**DOI:** 10.7759/cureus.38766

**Published:** 2023-05-09

**Authors:** Mohamed Kaakoua, Aboubaker Boufdil, Mohammed El Fadli, Rhizlane Belbaraka, Ismail Essadi

**Affiliations:** 1 Department of Medical Oncology, Ibn Sina Military Hospital, Marrakesh, MAR; 2 Department of Medical Oncology, Mohammed VI University Hospital Center, Marrakesh, MAR

**Keywords:** pubmed, medical publications, bibliometric study, morocco, medical oncology

## Abstract

Medical publications constitute an essential tool for sharing scientific advances in the medical field. They are also an educational tool of great significance in initial medical training, and also in further medical education. These publications are necessary to ensure an interface between the researchers and members of the medical scientific community, who are always looking for the correct and optimal treatment for their patients. Several golden rules have been laid out in terms of assessing the improvement in scientific productivity, namely the quality of the subject, the type of publication, the publication review and its impact factor, and the establishment of international collaborations.

Bibliometrics is a quantitative and qualitative analysis of scientific publications, which aids in assessing the scientific productivity of a community or a scientific institution. To the best of our knowledge, this is the first bibliometric study to evaluate the scientific productivity in the field of medical oncology in Morocco.

## Introduction and background

Medical oncology in Morocco is a young specialty that faced many obstacles when it was first instituted in 2004. However, it has managed to overcome these obstacles and hurdles thanks to the creation of the Moroccan Association for Training and Research in Medical Oncology (AMFROM). Currently, this specialty is attracting more and more attention from many Moroccan doctors.

The objective of this study was to analyze the contribution of Moroccan medical oncologists in the international medical literature and to discuss different ways to improve the quality and number of Moroccan publications in this emerging specialty.

## Review

Materials and methods

Bibliographic Research and Flowchart of the Study

A bibliometric and descriptive study was conducted based on a worldwide database (PubMed). As part of this study, we searched for all articles published in English or French and indexed in PubMed from January 2008 to December 2021. The combination of keywords included in this study was "medical oncology" and "Morocco".

**Figure 1:**
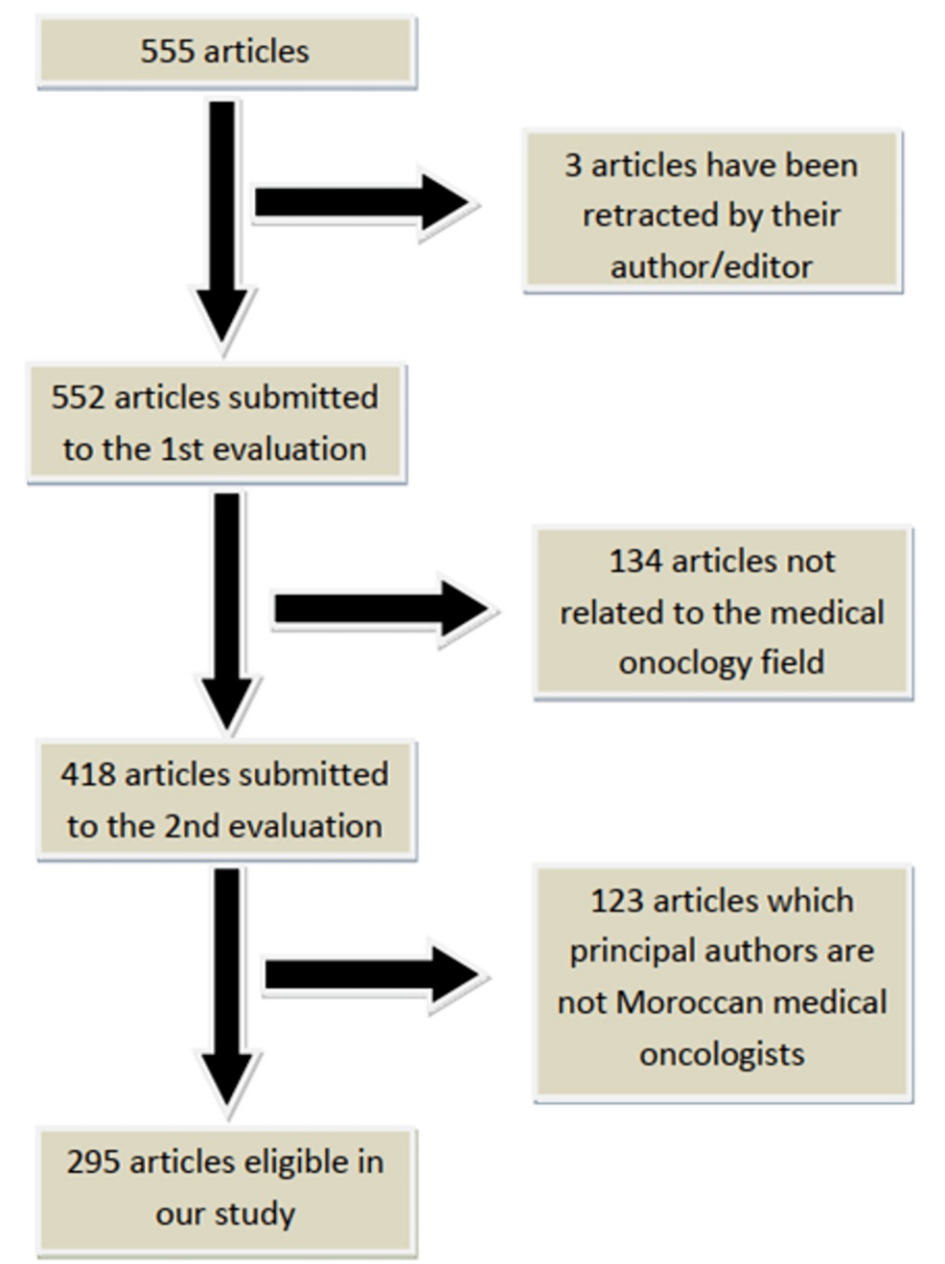
Flowchart depicting study selection and design

In this study, articles whose topics were not related to the medical oncology field were excluded after a meticulous analysis of the article’s title and abstract. Then, we excluded the articles whose principal authors (the first author or the last one) are not Moroccan medical oncologists (Figure 1).

Data Extraction and Analysis

A total of 555 articles from the PubMed database were analyzed, and 295 articles that met the inclusion criteria were selected. Several parameters were analyzed in our study, in particular the type of article (clinical case, literature review, prospective study, retrospective study, abstract), the hospital from which the article originated, the city where the hospital is located, the article's language of publication, the year of publication, the journal of publication and its type (oncological or non-oncological), the location of the tumor discussed in the article, and the presence or absence of collaboration and its type (international, national, local, or none).

To assess the journal's quality, we used the impact factor (IF) indicator, which calculates the average number of journal citations over the last two years preceding the year of this study [1]. The IF of each journal was identified using the website https://www.scijournal.org/. The authors of these articles were classified according to their H-index. To calculate this indicator, we used the website https://www.scopus.com/.

Results

The articles were evaluated using standard criteria, such as the year of publication, publication city, publication language, type, topics, collaboration status, IF, and H-index.

Publication by Year

**Figure 2:**
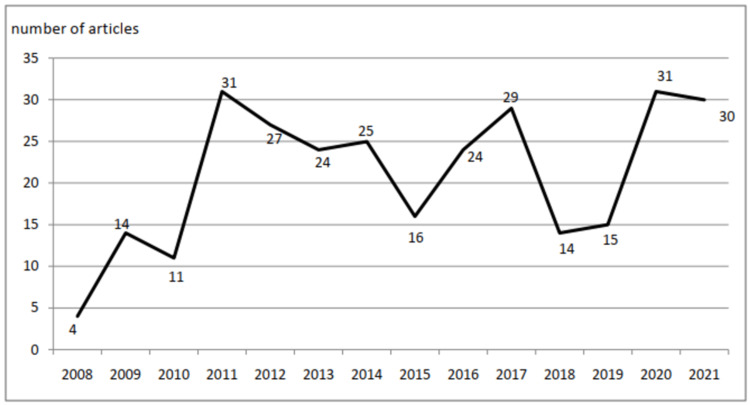
The evolution of the number of articles published (2008–2021)

A total of 295 Moroccan articles met the criteria of our study. In 2008, which corresponds to the year of the beginning of the study, only four articles were published by Moroccan medical oncologists. This number increased in 2011 to reach 31 published articles. However, this positive trend was interrupted in the following years, with only 16 articles published in 2015. There was a subsequent increase over the following two years, with a total of 29 articles published in 2017, before seeing another decrease in 2018 with only 14 published that year. Finally, the last year of our study showed the contribution of 30 articles by Moroccan medical oncologists (Figure 2).

Publication by City

Of the 295 Moroccan articles analyzed in our study, two cities contributed 75.25% of these articles. Rabat and Fez contributed 162 (54.91%) and 60 articles (20.33%), respectively. The three most productive institutions that contributed articles to our study are as follows: the National Institute of Oncology (NIO), located in Rabat, with 137 articles (46.44%), followed by the Hassan II University Hospital, located in Fez, with 60 (20.33%), and finally, in third place, the Mohammed V Military Hospital, located in Rabat, and the Mohammed VI University Hospital, located in Oujda, with 25 articles each (8.47%).

Type of Publication

**Figure 3:**
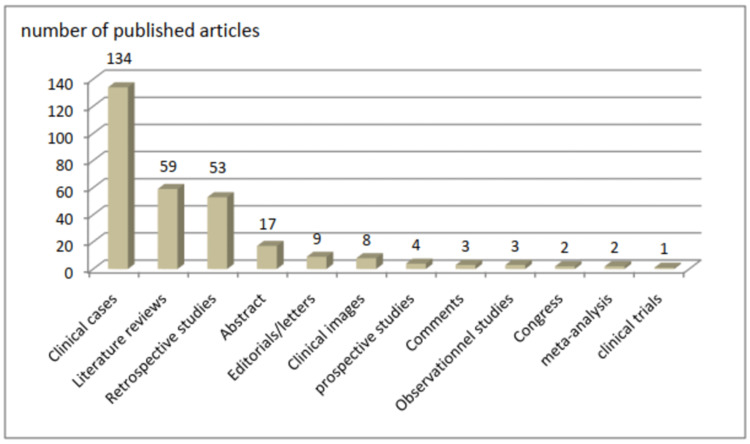
Type of articles published

Nearly half of the articles were published as clinical cases (134 articles), followed by 59 literature reviews (20%) and 53 retrospective studies (17,96%). It is important to mention that only two meta-analyses were found among the articles (0.67%). Figure 3 shows this distribution. Regarding the type of journal, the majority of articles were published in oncology journals (150 articles; 50.8%), while the rest were published in non-oncology journals (145 articles; 49.2%).

Publication by Topic

Breast cancer was the most discussed topic in the articles relevant to our study (49 articles; 16%), followed by lung cancer in the second position (27 articles; 9%) and sarcoma in the third position (26 articles; 8.8%).

Collaboration and Language of Publication

Institutional collaboration was noted in 201 articles (68.13%). This type of collaboration was found to be local in 122 articles (41.35%), national in 48 (16.27%), and only 31 articles (10.5%) involved international collaboration. Regarding the language of publication of these articles, English was by far the most used language (275 articles; 93.33%), while the French language represented only 6.77% (20 articles).

Impact Factor and H-index

Of the 295 articles analyzed in our study, 34 articles were reported in nine journals with an IF ≥5. Twenty-three articles in 14 journals had an IF ≤2 but <5, 53 articles in 18 journals had an IF <2, and 185 articles were published in 44 journals with unrecorded IF. The journals with the most number of articles relevant to our study are as follows: Pan African Medical Journal with 43 articles (IF unrecorded), Journal of Medical Case Reports with 20 articles (IF unrecorded), and Annals of Oncology with 17 articles (IF=10,575). It is worth mentioning that only one article was published in the prestigious New England Journal of Medicine (NEJM), with an IF of 37,909.

Our study showed the involvement of a total of 101 Moroccan medical oncologists, with 66 of them having an H-index <5. Forty-two Moroccan authors were affiliated with the NIO of Rabat, and 26 authors were affiliated with the Hassan II University Hospital of Fez. The average H-index was 4.46, with the highest H-index of 21 achieved by only one author.

Discussion

**Figure 4:**
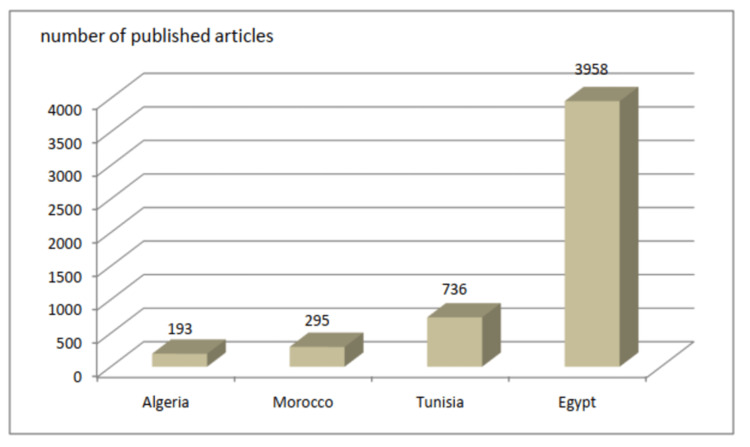
Comparison of the total number of articles by Moroccan oncologists with that from three other North African countries (Algeria, Tunisia, and Egypt)

The results of our study have identified a total of 295 articles produced by Moroccan medical oncologists during the period from 2008 to 2021. Publication-related productivity was inconsistent and variable, ranging from a minimum of four articles to a maximum of 31 articles per year. These results are relatively satisfactory if we take into account the fact that medical oncology is a new and emerging specialty in Morocco with a small number of medical oncology departments currently operational in the country. However, when we used the data on the website (http://www.scimagojr.com) to compare Morocco with other North African countries (Algeria, Tunisia, and Egypt), which share roughly the same socioeconomic conditions, it has become clear that Moroccan medical oncologists have a lot of work to do. During the same period, Algeria had 193 publications, Tunisia 736, and Egypt 3958 (Figure 4).

**Figure 5:**
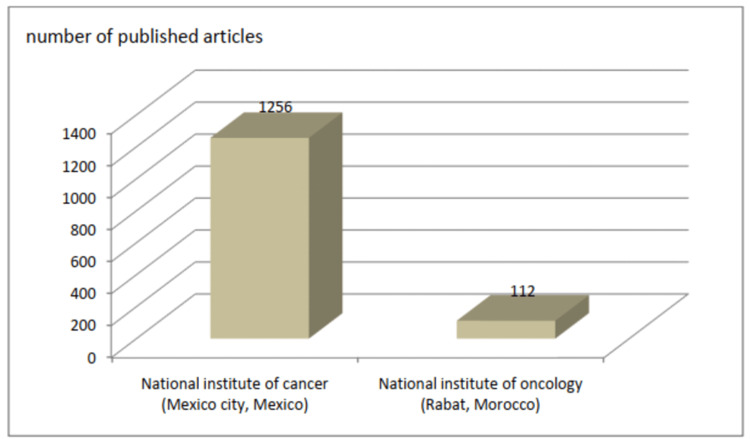
Comparison of the total number of articles between the NIO of Rabat and the National Institute of Cancer in Mexico City

Another interesting finding is the significantly high contribution of medical oncologists associated with the cities of Rabat and Fez (75,25% of articles), with the NIO of Rabat contributing almost half of the articles in our study (46,44%). This high publication rate, especially from the NIO, can be attributed to the fact that NIO was the first Moroccan institute to employ medical oncologists and provide residency training to young Moroccan medical oncologists. However, compared to cancer institutes in other countries, the number of publications is still quite low. Figure 5 shows a comparison between the scientific production of the National Cancer Institute in the capital of Mexico (Mexico City) during the period from 2008 to 2017 and that of the NIO during the same period [2].

Regarding article types, most of the articles were in the form of clinical cases, retrospective studies, and literature reviews (83.38%). These types of articles are associated with low values with regard to evidence-based medicine (EBM) and should be replaced by meta-analyses and randomized studies with high EBM [3,4]. These results are very poor in comparison to those obtained in the bibliometric study conducted in Iran, which collected 24,867 oncology-related articles over a period of 44 years. This study revealed that over 92% of the published articles were original research articles [5]. However, conducting such complicated research requires overcoming many barriers [6], including:

- The need for an information database that can save a huge amount of data to ensure long-term follow-up of cancer patients.

- The absence of an updated national cancer registry.

- The need for structured plans in conducting research.

- Lack of interest in research among young Moroccan medical oncologists, who view research only as a means to achieve their training objectives.

It is important to mention that one of the most difficult obstacles to overcome in Morocco is the lack of human resources, especially financial resources. This obstacle has been highlighted by the American Society of Clinical Oncology (ASCO), which concluded that a lack of financial resources is a serious impediment to conducting research among medical oncologists from underdeveloped and developing countries [7].

Breast cancer was the most discussed cancer type in the articles relevant to our study, which may be related to its high incidence in Morocco, accounting for 20% of the new cancer cases in the region of Casablanca according to the cancer register of Casablanca from 2008 to 2012 [8]. A similar trend was observed in a bibliometric study conducted by Iranian medical oncologists, which concluded that breast cancer was the most discussed cancer type in Iranian articles [5].

The majority of published articles (93.33%) are written in English. This is a significant number for a country like Morocco, where teaching at the faculty level is mostly done in French. On a global scale, English remains an essential language in the medical field, according to a major study [9]. This is because all international conferences and prestigious journals with high IF use English as the predominant medium of communication and publication.

The international and national collaborations are still weaker (26.77%) in comparison with the local ones (41.35%). Meanwhile, 31.86% of the articles are without any kind of collaboration. This is probably due to the absence of university exchange programs provided by the Moroccan government to increase cooperation between Moroccan medical oncologists and their colleagues from foreign countries. Thus, most international collaborations are restricted to university professors who already have contact with their foreign counterparts. In addition, the relevance of research in Morocco is still problematic with most projects lacking attractiveness, thereby making international cooperation difficult to find.

The majority of the articles included in our study were published in journals with an unrecorded IF (185 articles), and half of these articles were in non-oncology journals. This was a disappointing finding because scientific productivity is strongly related to publishing in prestigious journals with high IF. Thus, targeting these prestigious journals should be a priority for Moroccan medical oncologists to increase the scientific production of institutions. Furthermore, publishing in these kinds of journals will guarantee a high citation count for Moroccan authors and, consequently, a higher H-index. This can probably explain the low average of the H-index found in our study (H-index=4.46). The author with the highest H-index in Morocco has an H-index of 21, while it is 33 in Egypt and 13 in Tunisia.

From an International perspective, the ASCO has suggested some measures that would help countries increase their scientific productivity, which has been disrupted significantly during the COVID-19 pandemic [7] [10]. To increase scientific productivity in Morocco, various measures should be implemented, including:

- Providing well-balanced financial support across all the regions of the Kingdom.

- Developing a research network that will ensure national and international cooperation.

- Providing personalized supervision for all healthcare workers in the research field.

- Creating a Moroccan-indexed journal where the work of young Moroccan medical oncologists will be published. It will be a source of motivation for them to publish more works.

- Making clinical research more accessible, affordable, and equitable.

- Creating more pragmatic clinical experiments.

- Facilitating legislative and administrative policies to overcome limitations in the conduct of research.

- Recruiting and supervising healthcare workers in the field of research [11].

- Ensuring a reliable evaluation of the conduct of clinical trials and their results [12].

Limitations

Our analysis has some limitations. Initially, the screening of 555 articles was done manually, which may have led to several errors occurring during article screening and analysis. Secondly, we only included articles in which the first or last author was a Moroccan medical oncologist. Moreover, all articles that were not open-access, full-text, or written in English or French ​​were excluded from the search.

## Conclusions

Research is an extremely complex process the success of which depends mainly on the environment in which the study is conducted. The more favorable the conditions are in terms of human resources, financial resources, motivation, and supervision, the more satisfactory the results are likely to be. Our study has shown that Moroccan medical oncologists have the potential to increase their scientific productivity, which would enhance the country's overall productivity as well. To achieve this, they must be encouraged, supported, and assisted by the concerned authorities by implementing the measures and policies that we have proposed.
